# Male with Altered Mental Status

**DOI:** 10.5811/cpcem.2020.12.50960

**Published:** 2021-01-26

**Authors:** Jason Kondrat, Ben Ilyaguyev, Jonathan Stern, Teresa Choe, Josh Greenstein, Barry Hahn

**Affiliations:** * Staten Island University Hospital, Department of Emergency Medicine, Staten Island, New York; † Staten Island University Hospital, Department of Radiology, Staten Island, New York

**Keywords:** confusion, magnetic resonance imaging

## Abstract

**Case Presentation:**

A 62-year-old male presented to the emergency department with altered mental status and fever. Computed tomography of the head showed enlargement of the left lateral ventricle. Magnetic resonance imaging demonstrated debris and purulence in the ventricle along with edema and transependymal flow of cerebrospinal fluid surrounding both ventricles.

**Discussion:**

The patient was diagnosed with ventriculitis. Ventriculitis is an uncommon but serious disease. Early recognition and treatment are essential.

## CASE PRESENTATION

**Image 1:**
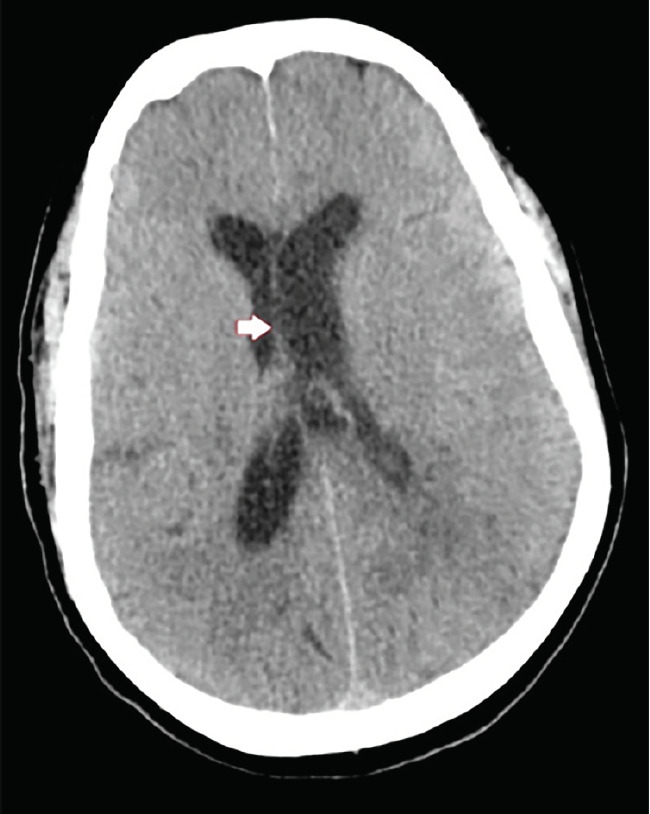
Axial computed tomography of the head with intravenous contrast showing mild enlargement of the left lateral ventricle (arrow).

**Image 2:**
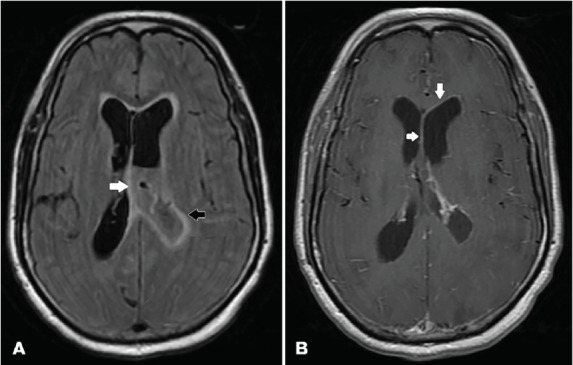
A) Axial magnetic resonance imaging (MRI) (fluid-attenuated inversion recovery sequence) showing mild enlargement of the left lateral ventricle, with debris and purulence in the ventricle (white arrow). Mild edema and transependymal flow of cerebrospinal fluid surrounding both ventricles are present (black arrow). B) Axial MRI without contrast showing enhancement of the left ventricle (white arrows).

A 62-year-old male with no significant past medical history presented to the emergency department with altered mental status for one day. Temperature was 103.3° degrees Fahrenheit, and remaining vital signs were within normal limits. He was alert to person and place only and was not responding appropriately to most questions or commands. Cranial nerves were intact, and upper and lower extremity strength was grossly normal and symmetrical. He resisted flexion of his neck. The patient was empirically started on vancomycin, ampicillin, ceftriaxone, and dexamethasone. Computed tomography (CT) of the head and magnetic resonance imaging (MRI) were performed (Images 1 and 2).

Lumbar puncture obtained cerebrospinal fluid (CSF) with 7628 nucleated cells per microliter (mL) (reference range: 0–5 cells/mL); 83% neutrophils (0–3%); glucose of 4 milligrams per deciliter (mg/dL) (45–75 mg/dL); and protein of 68 mg/dL (15–45 mg/dL).

## DISCUSSION

Ventriculitis is an inflammation of the ependymal lining of the cerebral ventricles, usually secondary to infection or trauma. Ventriculitis has no accepted diagnostic criteria. Meningitis, cerebral abscess with intraventricular rupture, ventricular catheters and shunts, neurosurgical complications, intrathecal chemotherapy, and trauma are potential causes.^1^ However, malignancy should also be considered in the differential diagnosis. Neisseria meningitidis and skin flora are the common causative agents.^1^–^3^ Gram-positive bacteria are most common in infections involving ventricular implants such as shunts. Gram-negative bacteria are a consideration in postoperative neurosurgical ventriculitis. Ventriculitis secondary to ventricular catheters and shunt infection has an incidence of 10% and varies depending on insertion technique and management. Ventriculitis secondary to meningitis is more commonly seen in infants and immunocompromised individuals.^1^

Symptoms classically include fever and meningismus. Investigations for ventriculitis include CSF sampling and imaging. Cerebrospinal fluid sampling and neuroimaging are vital in making the diagnosis. Cerebral spinal fluid protein greater than 50 mg/dL, glucose less than 25 mg/dL, pleocytosis with greater than 10 cells/mL, and greater than 50% polymorphonuclear neutrophils are suggestive of ventriculitis. Cultures may be negative, despite active infection. The Infectious Diseases Society of America recommends MRI as the modality of choice.^3^ Characteristic MRI findings include intraventricular debris and pus, abnormal periventricular and subependymal signal intensity, and enhancement of the ventricular lining. Non-contrast CT most frequently demonstrates dependent, hyperdense layering material, particularly in the occipital horns of the lateral ventricles. Hydrocephalus and periventricular low density may also be present. With contrast CT, enhancement of the ependymal lining of the ventricles may be seen.

**CPC-EM Capsule**

What do we already know about this clinical entity?

Ventriculitis is an inflammation of the ependymal lining of the cerebral ventricles, usually secondary to infection or trauma.

What is the major impact of the image(s)?

The diagnosis of ventriculitis is both interesting and rare. Early recognition and treatment are essential in treating this serious disease.

How might this improve emergency medicine practice?

Maintaining this disease process in the differential diagnosis lessens the potential for significant morbidity and mortality. Early recognition is essential.

The mainstay of treatment is intravenous antibiotics and removing any devices for catheter-related infection. Initially, empirical therapy is used based on the patient’s age and etiology. For catheter-related ventriculitis, this is generally vancomycin and an anti-pseudomonal beta-lactam. Purulent material due to ventriculitis may result in occlusion of the CSF pathways, causing obstructive or multiloculated hydrocephalus. Mortality rates vary from 10–75%, but quality studies evaluating prognosis are lacking.^4^

Our patient was admitted to the medical ward for sepsis and ventriculitis. Both neurology and infectious disease consults were obtained. On the second day of hospitalization the patient became obtunded and underwent endotracheal intubation for airway protection. He was subsequently upgraded to the intensive care unit. The patient continued to deteriorate despite optimal care and expired on hospital day five.
